# The prevalence and metabolic characteristics of polycystic ovary syndrome in the Qatari population

**DOI:** 10.1371/journal.pone.0181467

**Published:** 2017-07-19

**Authors:** Soha R. Dargham, Lina Ahmed, Eric S. Kilpatrick, Stephen L. Atkin

**Affiliations:** 1 Infectious Disease Epidemiology Group, Weill Cornell Medicine, Doha, Qatar; 2 Research Faculty, Weill Cornell Medicine, Doha, Qatar; 3 Department of Pathology, Sidra Medical and Research Center, Doha, Qatar; University of Colorado Denver School of Medicine, UNITED STATES

## Abstract

**Objective:**

The prevalence of polycystic ovary syndrome (PCOS) in the Qatari population is unknown and hence the estimated impact on the local population cannot be determined. The purpose of this study was to estimate the prevalence and metabolic features of PCOS among Qatari women.

**Design:**

Cross sectional analysis.

**Patients:**

3,017 Qatari subjects volunteered to be phenotyped and genotyped for the Qatar Biobank from which all women between the ages of 18–40 years were identified (750).

**Measurements:**

720 women had testosterone and sex hormone binding globulin (SHBG) measurements. PCOS was diagnosed according the National Institute of Health (NIH) Guidelines of a raised androgen level (free androgen index >4.5 or a raised total testosterone) and menstrual irregularity after the exclusion of other conditions.

**Results:**

All results are reported as mean value of PCOS versus control. 87 of 720 women fulfilled the NIH guidelines (12.1%) for PCOS specifically using a free androgen index greater than 4.5 or a total testosterone greater than 2.7nmol/l and menstrual irregularity. Subjects were heavier with a more metabolic profile of a greater systolic and diastolic blood pressure, higher levels of C reactive protein, insulin (p<0.01) and HbA1c (P<0.02), and decreased HDL levels (p<0.01). Pulse wave velocity as a marker of arterial stiffness was also increased (p<0.05)

**Conclusions:**

By NIH guidelines the prevalence of PCOS in this Qatari cohort was 12.1% that would likely reflect 20% by Rotterdam criteria, with a markedly more metabolic phenotype than Qatari controls.

## Introduction

Polycystic ovary syndrome (PCOS) is one of the most common endocrine disorders and affects 6–20% of reproductive-aged women[1–3] that leads to irregular periods, infertility and increased androgen levels causing hirsutism and acne[4, 5]. Obesity affects the majority of women with PCOS, and they have a higher prevalence of both impaired glucose tolerance and type 2 diabetes[3]. Women with PCOS show increased cardiovascular risk through a higher incidence of hypertension, an adverse lipid profile, and insulin resistance (IR)[6, 7]. The underlying pathophysiology of PCOS is unclear and appears to be a combination of hyperandrogenism[8], insulin resistance and the factors that cause follicular arrest[9]. Up to 60% of PCOS women have insulin resistance, up to 40% have impaired glucose tolerance and 10% may develop type 2 diabetes by the age of 40 years[9]. Whilst both slim and obese women with PCOS have insulin resistance, the development of obesity may exacerbate the phenotype leading to increased cardiovascular risk[10]. In addition, insulin resistance appears to have both direct and indirect effects on the androgen excess in PCOS[9]. In a recent systematic analysis suggests that the prevalence of PCOS was put at between 6% (NIH criteria) to 10% (Rotterdam and Androgen excess society guidelines)[11]. Differing diagnostic criteria have contributed to the confusion regarding prevalence; for example, use of the Rotterdam criteria led to over twice the prevalence compared to that of the National Institute of Health (NIH) criteria for the diagnosis of PCOS[11]. The prevalence of PCOS also differs according to ethnic background; for example, women from South East Asia often present at a younger age resulting from a more severe phenotype leading to more severe symptoms[12, 13]. From *in silico* analysis of the phenotype-genotype relationship using single nucleotide polymorphisms to look at the degree of genetic similarity it is predicted that women of the Middle East will have a more severe hyperandrogenic phenotype[14]. A recent study in a very small cohort of Qatari women suggested that the prevalence of PCOS was 18.3% that was remarkably high[15]. This study has used the much larger Qatar Biobank to establish a more robust prevalence for PCOS in the country.

## Materials and methods

The Qatar biobank (QBB) is a large-scale, long term medical research initiative for the population of Qatar, which over the next few years aims to recruit 60,000 men and women Qatari nationals and long-term residents (>15 years residence) aged ≥18 years, and to follow up these same individuals over the long term to record any subsequent health conditions. At the time of this study a total of 4500 subjects had been recruited aged between 18 and 89 years of age. At the baseline recruitment visit, extensive clinical phenotypic information is collected from each participant. Collection of the demographic, biochemical and genetic data were approved by the QBB IRB and by the Ministry of Health Qatar and all subjects gave their informed consent (http://www.qatarbiobank.org.qa). Subjects are recruited by advert and by word of mouth from those attending the QBB, in addition to referral to the QBB for those with out of range values from Hamad Hospital the majority of which are for abnormal bone density 46.5%, dyslipidemia 39.2% and hypertension 11.6%. All participants give written informed consent. This was a cross sectional study involving 750 women between the ages of 18–40 inclusive were identified of whom 720 had complete information that included total testosterone, sex hormone binding globulin (SHBG): all had thyroid function tests, prolactin, dehydroepiandrosterone sulfate (DHEAS) and 17beta hydroxyprogesterone to exclude other confounding diagnoses. The diagnosis of PCOS for the comparison with the QBB was based on the NIH criteria of biochemical evidence of hyperandrogenemia (free androgen index >4.5) or a raised testosterone greater than 2.7nmol/l, and oligomenorrhea or amenorrhea. All identified PCOS subjects had no documented concurrent illness and were not on any medication. All of the control women had regular periods, no biochemical hyperandrogenemia and no documented medical history nor were taking any medications. Though a medical history was captured specific questions on hirsutism, acne and alogenic alopecia were not asked and therefore this data were not captured robustly.

Height, weight and waist circumference and body mass index (BMI) were performed according to WHO guidelines[16]. Pulse Wave Velocity (PMV) was measured using VICORDER^®^ PC 400 300E (SMT medical GmbH & Co. KG, Wuerzburg, Germany). The participants were asked to lie on the bed in a 30o angle position with arms and hands resting at their sides. Cuffs were applied tightly to the participant’s upper right arm and upper right thigh. The distance from the top of the arm cuff to the top of the thigh cuff in a straight line with the arms by the participants’ side was recorded prior to PMV measurement by the Vicorder.

The entire study was performed in Qatar with subjects presenting to the QBB and with all of the samples being processed and analysed at Hamad Medical Corporation, Qatar.

### Collection and analysis of blood samples

Blood samples were collected and immediately processed (within 5 min) and stored frozen at -80°C pending analysis. TSH, prolactin, insulin, testosterone, C reactive protein (CRP), DHEAS, and SHBG were conducted in the Chemistry Laboratory at Hamad Medical Corporation, Doha, Qatar, and measured by an immunometric assay with fluorescence detection on the DPC Immulite 2000 analyzer using the manufacturer’s recommended protocol. The Abbott testosterone method was performed within manufacturer’s specification throughout the sample collection (within run coefficient of variation (CV) 3.1%, within laboratory CV 3.6% at 2.6nmol/L). The free androgen index (FAI) was calculated as the total testosterone x 100/SHBG. Serum insulin was assayed using a competitive chemiluminescent immunoassay performed on the manufacturer’s DPC Immulite 2000 analyzer (Euro/DPC, Llanberis, UK). The analytical sensitivity of the insulin assay was 2μU/ml, the coefficient of variation was 6%, and there was no stated cross-reactivity with proinsulin. Plasma glucose was measured using a Synchron LX 20 analyzer (Beckman-Coulter), using the manufacturer’s recommended protocol. The coefficient of variation for the assay was 1.2% at a mean glucose value of 5.3 mmol/L during the study period. The insulin resistance was calculated using the HOMA method [HOMA-IR = (insulin x glucose)/22.5]. Data were supplied to Weill Cornell Medicine Qatar biostatistics unit from the QBB in an anonymous coded manner that had been approved by the Weill Cornell Medicine Qatar IRB.

### Statistical analysis

The biostatistics unit at Weill Cornell calculated the sample size needed to detect a PCOS prevalence of 10% (equivalent to a Caucasian population) among the Biobank volunteer Qatari women assuming a standard normal variate of 1.96 for a significance level of 95% (type I error α = 0.05), and assuming that the hypothetical prevalence of the condition to be detected is 10%, then estimate of the minimum sample size needed at a precision level of 3% would be 385 women.

Data trends were visually and statistically evaluated for normality. Non-parametric tests (Mann Whitney U) were applied on data that violated the assumptions of normality when tested using the Kolmogorov-Smirnov Test. Statistical analysis was performed using SPSS for Windows, version 24.0. All values are given as (mean ± SD) unless specified. All values are given as mean and 95% confidence interval (CI) unless specified.

## Results

The demographics of women with PCOS and controls are shown in Table 1. 97 of 720 women fulfilled the NIH guidelines (12.1%) for PCOS specifically using a free androgen index greater than 4.5 ((testosterone/SHBG) x 100), or an elevated isolated total testosterone greater than 2.7nmol/l and menstrual irregularity. These data would estimate a prevalence of 20% by Rotterdam/Androgen Society criteria by extrapolation[11]. PCOS subjects were younger than control subjects (p = 0.02). In accord with the recognized phenotypic features, PCOS subjects were heavier with an increased BMI (p<0.01) and waist circumference was greater (p<0.01), systolic and diastolic blood pressure were higher (p<0.01). Vicorder pulse wave velocity was greater, indicative of increased arterial stiffness (p = 0.04). CRP indicative of inflammation and insulin were elevated in the PCOS group (p<0.01), as was HbA1c (p<0.01). HDL levels were decreased (p<0.01); however, LDL levels did not differ (p = 0.55) nor did glucose levels (p = 0.55).

**Table 1:** Demographic details for 720 women between the ages of 18 to 40 years, comparing normal subjects with women defined as having polycystic ovary syndrome by NIH criteria (free androgen index greater than 4.5, with irregular menses).

	Control n = 633	PCOS n = 87	P-value
	Mean (95% CI)	Mean (95% CI)	
**Age (years)**	29.25 (28.35–30.15)	26.88 (24.62–29.14)	0.02
**Weight kg**	68.47 (65.87–71.08)	79.33 (73.05–85.62)	<0.01
**BMI**	26.87 (26.38–27.36)	31.60 (29.11–34.08)	<0.01
**Waist cm**	78.81 (76.92–80.70)	89.28 (83.97–94.59)	<0.01
**Blood Pressure—Systolic mm/Hg**	104.57 (103.05–106.08)	110.20 (105.45–114.95)	<0.01
**Blood Pressure—Diastolic mm/Hg**	70.79 (69.60–71.98)	75.32 (72.15–78.49)	<0.01
**HBA 1C %**	5.36 (5.25–5.47)	5.58 (5.22–5.95)	<0.01
**Glucose**	4.99 (4.81–5.18)	4.85 (4.47–5.22)	0.73
**Insulin (μIU/ml)**	15.10 (7.74–22.46)	16.28 (12.46–20.10)	<0.01
**Insulin Resistance (HOMA)**	3.63 (2.68–4.58)	4.03 (3.00–5.06)	<0.01
**Cholesterol mmol/l**	4.72 (4.60–4.83)	4.69 (4.35–5.03)	0.16
**High Density Lipoprotein mmol/l**	1.51 (1.46–1.56)	1.32 (1.21–1.43)	<0.01
**Low Density Lipoprotein mmol/l**	2.78 (2.68–2.89)	2.78 (2.46–3.09)	0.55
**Testosterone—nmol/l**	1.13 (1.06–1.21)	1.93 (1.71–2.15)	<0.01
**C-Reactive Protein—mmol/dl**	6.49 (5.82–7.17)	7.08 (5.02–9.14)	<0.01
**Vicorder—Pulse Wave Velocity**	9.88 (9.41–10.34)	10.52 (9.62–11.42)	0.04

## Discussion

Using the NIH guidelines the initial prevalence in this cohort was 12.1% by the NIH criteria that may translate into 20% using either the Rotterdam or Androgen Excess Society criteria[11]. This is much higher than that suggested by the recent systematic review that suggested that the prevalence was between 6% (NIH criteria) to 10% (Rotterdam and Androgen excess society guidelines) for Caucasian subjects [11]. This is lower than the Qatari study that suggested that the prevalence was 18.3% though this latter study included women with clinical hyperandrogenism and may have overestimated the prevalence through the small sample size and the method of recruitement[15]. Clinical hyperandrogenism defined by the Ferriman and Gallwey score is not validated in this ethnic population and the cut off of a value greater than 8 may not be appropriate, and others have used a cut off value of over 17 in the Middle East[15] [17]. For this reason inclusion of clinical hirsutism reported in the clinical data was not used in this study.

A hyperandrogenemic metabolic phenotype for these women with PCOS from this geographical location would be predicted from the literature [14], and that appeared to be the case. PCOS patients were heavier, with significantly higher insulin, insulin resistance and testosterone values. They appeared to have an adverse metabolic phenotype with higher systolic and diastolic blood pressures, and lower HDL levels[6]. These metabolic findings were compounded by an increased CRP that is a measure of inflammation and an independent risk factor for CVD[18]. Pulse wave velocity is a marker of arterial stiffness that correlates well to cardiovascular risk and was increased in the PCOS group. In young subjects (reported mean age 31 years) it has been suggested that this increased arterial stiffness may relate to greater blood pressure variability [19] that may in turn reflect increased cardiovascular risk [20]. These differences in PCOS prevalence across differing populations are likely are not just due to definitions, but rather due to differences in ethnicity as well as changes in awareness, lifestyle and obesity rates as well. Of note, over 61% of the 749 women identified were either overweight (BMI 26–29.9; 31.4%) or obese (BMI greater than 30; 29.7%). It is clear that obesity per se increases cardiovascular risk factors independently of PCOS(10), but that the inherent insulin resistance found in may PCOS patients adds to the cardiovascular risk burden(9), particularly with the development of type 2 diabetes. In this Qatari population, whilst statistically very significant due to the number of subjects, in real terms each of the parameters of the increased insulin levels, insulin resistance, blood pressure, HbA1c, hsCRP, PWV and lower HDL will likely be of minimal clinical significance in this age group and indeed when the parameters are entered into the Framingham equation there is no excess cardiovascular risk compared to control subjects. However, these factors may translate into greater risk later in life and this group of subjects needs to be compared to an older group of women to determine if this combination of parameters may reflect increased cardiovascular disease.

The strength of this study was the power to be able to ascertain the prevalence of PCOS by the NIH criteria. The power calculation was based on 10% prevalence 3% precision with 80% power; however, as higher prevalence was found than expected then at 14% or 20% prevalence with the same precision of 3% it would need 514 or 683 patients, respectively; therefore, still within the power of the study. Limitations of the study included that ovarian ultrasound was not performed precluding assessment according to the Rotterdam and Androgen Excess Society criteria. The referral to the QBB could be a source of bias as the recruitment from secondary care was from those subjects with ab parameters of bone densitometry, frank hypertension or abnormal lipids; however, those parameters did not affect any of those in the PCOS population. Potential bias from word of mouth recruiting family members could have been an issue given the estimated 78% heritability for PCOS[21].

In summary, using the NIH guidelines the initial prevalence in this cohort was 12.1%, and data suggests that this would translate into a prevalence of approximately 20% using the Rotterdam/Androgen Society criteria for PCOS. The Qatari PCOS group had a more metabolic phenotype than control subjects.

## References

[1] Yildiz BO, Bozdag G, Yapici Z, Esinler I, Yarali H. Prevalence, phenotype and cardiometabolic risk of polycystic ovary syndrome under different diagnostic criteria. Human reproduction. 2012;27(10):3067–73. doi: 10.1093/humrep/des232 22777527 10.1093/humrep/des232

[2] Teede HJ, Joham AE, Paul E, Moran LJ, Loxton D, Jolley D, et al Longitudinal weight gain in women identified with polycystic ovary syndrome: results of an observational study in young women. Obesity (Silver Spring). 2013;21(8):1526–32. doi: 10.1002/oby.20213 . 23818329 10.1002/oby.20213

[3] March WA, Moore VM, Willson KJ, Phillips DI, Norman RJ, Davies MJ. The prevalence of polycystic ovary syndrome in a community sample assessed under contrasting diagnostic criteria. Hum Reprod. 2010;25(2):544–51. Epub 2009/11/17. doi: 10.1093/humrep/dep399 . 19910321 10.1093/humrep/dep399

[4] Norman RJ, Dewailly D, Legro RS, Hickey TE. Polycystic ovary syndrome. Lancet. 2007;370(9588):685–97. Epub 2007/08/28. doi: 10.1016/S0140-6736(07)61345-2 . 17720020 10.1016/S0140-6736(07)61345-2

[5] Ehrmann DA. Polycystic ovary syndrome. N Engl J Med. 2005;352(12):1223–36. Epub 2005/03/25. doi: 10.1056/NEJMra041536 . 15788499 10.1056/NEJMra041536

[6] Sathyapalan T, Atkin S. REVIEW TOPIC ON MECHANISMS IN ENDOCRINOLOGY Recent Advances in the Cardiovascular Aspects of Polycystic Ovary Syndrome. European journal of endocrinology / European Federation of Endocrine Societies. 2011 Epub 2011/11/19. doi: 10.1530/EJE-11-0755 .22096112

[7] Moran L, Teede H. Metabolic features of the reproductive phenotypes of polycystic ovary syndrome. Hum Reprod Update. 2009;15(4):477–88. Epub 2009/03/13. doi: 10.1093/humupd/dmp008 . 19279045 10.1093/humupd/dmp008

[8] Rosenfield RL, Ehrmann DA. The Pathogenesis of Polycystic Ovary Syndrome (PCOS). Endocr Rev. 2016:er20151104. Epub 2016/07/28. doi: 10.1210/er.2015-1104 . 27459230 10.1210/er.2015-1104 PMC5045492

[9] Dumesic DA, Akopians AL, Madrigal VK, Ramirez E, Margolis DJ, Sarma MK, et al Hyperandrogenism Accompanies Increased Intra-Abdominal Fat Storage In Normal Weight Polycystic Ovary Syndrome Women. J Clin Endocrinol Metab. 2016:jc20162586. Epub 2016/08/30. doi: 10.1210/jc.2016-2586 . 27571186 10.1210/jc.2016-2586 PMC5095243

[10] Kahal H, Aburima A, Ungvari T, Rigby AS, Dawson AJ, Coady AM, et al Polycystic ovary syndrome has no independent effect on vascular, inflammatory or thrombotic markers when matched for obesity. Clinical Endocrinology. 2013;79(2):252–8. doi: 10.1111/cen.12137. 23278130 10.1111/cen.12137

[11] Bozdag G, Mumusoglu S, Zengin D, Karabulut E, Yildiz BO. The prevalence and phenotypic features of polycystic ovary syndrome: a systematic review and meta-analysis. Hum Reprod. 2016;31(12):2841–55. Epub 2016/09/25. doi: 10.1093/humrep/dew218 . 27664216 10.1093/humrep/dew218

[12] Wijeyaratne CN, Balen AH, Barth JH, Belchetz PE. Clinical manifestations and insulin resistance (IR) in polycystic ovary syndrome (PCOS) among South Asians and Caucasians: is there a difference? Clinical endocrinology. 2002;57(3):343–50. . 12201826 10.1046/j.1365-2265.2002.01603.x

[13] Glintborg D, Mumm H, Hougaard D, Ravn P, Andersen M. Ethnic differences in Rotterdam criteria and metabolic risk factors in a multiethnic group of women with PCOS studied in Denmark. Clinical endocrinology. 2010;73(6):732–8. Epub 2010/09/18. doi: 10.1111/j.1365-2265.2010.03873.x . 20846294 10.1111/j.1365-2265.2010.03873.x

[14] Casarini L, Brigante G. The Polycystic Ovary Syndrome Evolutionary Paradox: a Genome-Wide Association Studies-Based, in silico, Evolutionary Explanation. J Clin Endocrinol Metab. 2014;99(11):E2412–20. Epub 2014/08/06. doi: 10.1210/jc.2014-2703 . 25093623 10.1210/jc.2014-2703

[15] Sharif E, Rahman S, Zia Y, Rizk NM. The frequency of polycystic ovary syndrome in young reproductive females in Qatar. Int J Womens Health. 2017;9:1–10. Epub 2016/12/30. doi: 10.2147/IJWH.S120027 ; 28031728 10.2147/IJWH.S120027 PMC5179205

[16] World, Health, Organization. Waist circumference and waist–hip ratio Report of a WHO expert consultation, Geneva 2008.

[17] Al-Ruhaily AD, Malabu UH, Sulimani RA. Hirsutism in Saudi females of reproductive age: a hospital-based study. Ann Saudi Med. 2008;28(1):28–32. Epub 2008/02/27. . 18299651 10.5144/0256-4947.2008.28 PMC6074238

[18] van Wissen S, Trip MD, Smilde TJ, de Graaf J, Stalenhoef AF, Kastelein JJ. Differential hs-CRP reduction in patients with familial hypercholesterolemia treated with aggressive or conventional statin therapy. Atherosclerosis. 2002;165(2):361–6. . 12417288 10.1016/s0021-9150(02)00280-0

[19] Boardman H, Lewandowski AJ, Lazdam M, Kenworthy Y, Whitworth P, Zwager CL, et al Aortic stiffness and blood pressure variability in young people: a multimodality investigation of central and peripheral vasculature. J Hypertens. 2017;35(3):513–22. Epub 2016/11/16. doi: 10.1097/HJH.0000000000001192 ; 27846043 10.1097/HJH.0000000000001192 PMC5278891

[20] Vishram JK, Dahlof B, Devereux RB, Ibsen H, Kjeldsen SE, Lindholm LH, et al Blood pressure variability predicts cardiovascular events independently of traditional cardiovascular risk factors and target organ damage: a LIFE substudy. J Hypertens. 2015;33(12):2422–30. doi: 10.1097/HJH.0000000000000739 . 26378687 10.1097/HJH.0000000000000739

[21] Dunaif A. Perspectives in Polycystic Ovary Syndrome: From Hair to Eternity. J Clin Endocrinol Metab. 2016;101(3):759–68. Epub 2016/02/26. doi: 10.1210/jc.2015-3780 . 26908109 10.1210/jc.2015-3780 PMC4803161

